# Maternal and Paternal Risk Factors for Cryptorchidism and Hypospadias: A Case–Control Study in Newborn Boys

**DOI:** 10.1289/ehp.7243

**Published:** 2004-09-03

**Authors:** Frank H. Pierik, Alex Burdorf, James A. Deddens, Rikard E. Juttmann, Rob F.A. Weber

**Affiliations:** ^1^Department of Andrology and; ^2^Department of Public Health, Erasmus MC, Rotterdam, the Netherlands; ^3^Department of Mathematical Sciences, University of Cincinnati, Cincinnati, Ohio, USA; ^4^Department of Child Health Care, Rotterdam Homecare Foundation, Rotterdam, the Netherlands

**Keywords:** children, cryptorchidism, endocrine disruptor, environment, epidemiology, hypospadias, nutrition, occupational exposure, testis

## Abstract

Little is known on environmental risk factors for cryptorchidism and hypospadias, which are among the most frequent congenital abnormalities. The aim of our study was to identify risk factors for cryptorchidism and hypospadias, with a focus on potential endocrine disruptors in parental diet and occupation. In a case–control study nested within a cohort of 8,698 male births, we compared 78 cryptorchidism cases and 56 hypospadias cases with 313 controls. The participation rate was 85% for cases and 68% for controls. Through interviews, information was collected on pregnancy aspects and personal characteristics, lifestyle, occupation, and dietary phytoestrogen intake of both parents. Occupational exposure to potential endocrine disruptors was classified based on self-reported exposure and ratings of occupational hygienists based on job descriptions. Our findings indicate that paternal pesticide exposure was associated with cryptorchidism [odds ratio (OR) = 3.8; 95% confidence interval (95% CI), 1.1–13.4]. Smoking of the father was associated with hypospadias (OR = 3.8; 95% CI, 1.8–8.2). Maternal occupational, dietary, and lifestyle exposures were not associated with either abnormality. Both abnormalities were associated with suboptimal maternal health, a lower maternal education, and a Turkish origin of the parents. Being small for gestational age was a risk factor for hypospadias, and preterm birth was a risk factor for cryptorchidism. Because paternal pesticide exposure was significantly associated with cryptorchidism and paternal smoking was associated with hypospadias in male offspring, paternal exposure should be included in further studies on cryptorchidism and hypospadias risk factors.

Cryptorchidism and hypospadias are among the most frequent congenital abnormalities in male births. Cryptorchidism (maldescent of the testis) is observed in 1–5% of full-term male births (Toppari et al. 1996) and is a risk factor for subfertility and testicular cancer. Hypospadias (abnormal location of the orifice of the urethra) is observed in 0.3–0.7% of male births and requires surgical treatment in most cases (Pierik et al. 2002).

In the past two decades, concern has been raised over a possible increase in disorders of the male reproductive tract, including cryptorchidism, hypospadias, testicular cancer, and impaired semen quality. It has been suggested that these disorders are interrelated and share a common etiology during fetal life, described by Skakkebaek and colleagues as the testicular dysgenesis syndrome (TDS) (Sharpe and Skakkebaek 1993; Skakkebaek et al. 2001). Fetal exposure to endocrine disruptors (EDs) with estrogen-like or antiandrogen-like activity has been suggested as a cause for TDS (Sharpe 2003; Sharpe and Skakkebaek 1993). Various groups of chemicals, including pesticides and phthalate esters, have been identified as being weakly estrogenic or antiandrogenic (Sharpe 2003). These chemicals may occur in working environments, drinking water, and food (Toppari et al. 1996). Humans can also be exposed to natural phytoestrogens, through consumption of food products derived from plants (Toppari et al. 1996).

There is only limited evidence that the suggested increase in male urogenital abnormalities in humans can be attributed to exposure to EDs (Sharpe 2003) or environmental chemicals in general. An excess of hypospadias has been reported among newborns in populations living within 2–3 km of landfill sites (Dolk et al. 1998; Elliott et al. 2001). These findings may indicate an effect of chemical wastes, but exposure classification was too crude to differentiate this exposure from confounding factors (Dolk et al. 1998; Elliott et al. 2001). In contrast, no association was observed between hypospadias and occupational exposure to EDs by the mother during pregnancy (Vrijheid et al. 2003). A maternal vegetarian diet during pregnancy has been associated with hypospadias in the offspring, suggesting a role of a higher intake of phytoestrogens (North and Golding 2000). Although several studies have demonstrated male-mediated developmental effects of environmental exposure (Davis et al. 1992; Robaire and Hales 2003), its role in the etiology of cryptorchidism and hypospadias remains unclear.

The aim of the present study was to evaluate the role of maternal and paternal occupational and dietary exposures to potential EDs in the occurrence of cryptorchidism and hypospadias.

## Materials and Methods

### Design and participants.

We conducted a nested case–control study within a large cohort of newborn boys in the city of Rotterdam. This cohort consisted of newborns who were examined at their first visit to child health care centers (CHCs). In the Netherlands, CHCs are notified of live births within 2 days after registration in the municipal birth register. CHCs invite all parents to participate free of charge in the nationwide preventive child health care program, including growth monitoring and vaccination. From 1 October 1999 to 31 December 2001, 9,146 male births were registered, of which 8,695 boys (95%) were examined by CHC physicians at a median age of 34 days (5th and 95th percentiles, 25 and 105 days, respectively). CHC physicians (*n* = 30) were trained in a standardized genital examination by a pediatric urologist and a pediatric endocrinologist during a workshop. In addition, all CHC physicians received written instruction on the genital examination procedure. During the course of the study, new CHC physicians were instructed on the standardized examination, and every 6 months a meeting with the CHC physicians, researchers, and expert pediatricians was organized to refresh the CHC physicians on the procedures. Boys were diagnosed as cryptorchid if one or both testes were nonpalpable or if they could not be manipulated to a stable position at the bottom of the scrotum (de Muinck Keizer-Schrama 1987). Hypospadias was defined as a displacement of the urethral meatus from the tip of the glans penis to the ventral side of the phallus, scrotum, or perineum (Pierik et al. 2002). All 91 cases of cryptorchidism (1.1%) and 67 cases of hypospadias (0.8%) that were identified by CHC physicians were eligible for the case–control study, of which four cases had both abnormalities. We selected controls from the 8,541 boys without cryptorchidism or hypospadias if their age was compatible with the observed age range of cases. For statistical power, three times more controls than cases were approached for participation.

Parents of cases and controls were invited to participate in the study, and after written informed consent a research nurse interviewed the mother with a structured questionnaire during a home visit approximately 11 weeks (median) after giving birth (5th and 95th percentiles, 6 and 27 weeks). If present, the father was also interviewed. This study was approved by the institutional review board. The participation rate among mothers was 86% (78 of 91) for cryptorchidism cases, 84% (56 of 67) for hypospadias cases, and 68% (313 of 462) for controls. This participation produced 443 mother–child pairs, including the four boys with both abnormalities. Paternal information was available for 326 of the 443 subjects (74%), in which the paternal information was provided by the biologic father in 91 subjects (28% overall, and 24 and 38% for controls and cases, respectively) and was filled out by the mother because of the father’s absence in 235 subjects (72%). The paternal questionnaire was considered a nonresponse when mothers could not provide core information on the biologic father regarding the country of origin of his parents, date of birth, or occupational history.

### Data collection.

A research nurse completed structured questionnaires during interviews with parents. Rotterdam is a multicultural city in which the main groups of immigrants originate from Turkey, Morocco, Surinam, and the Netherlands Antilles, the latter two being Dutch-speaking countries. When necessary because of language problems, a qualified interpreter read the questions aloud from translated written questionnaires in the Turkish or Moroccan-Arabic language. The maternal questionnaire gathered information on personal characteristics, health, pregnancy aspects, diet, and occupational history. The paternal questionnaire collected data on personal characteristics, health, and occupation. Personal characteristics were age, height, weight, education, country of origin, and lifestyle factors such as smoking habits and alcohol use during the past 12 months. Education level was defined as low (≤ 9 years), intermediate (10–14 years), or high (≥ 15 years). The country of origin of the mother and father was based on the country of birth of their parents (i.e., the newborn’s grandparents) as defined by Statistics Netherlands (Keij 2000). The country of origin assigned to foreigners (defined as someone with at least one parent born abroad) is that of the mother if both parents are born abroad; otherwise, it is the country of birth of the parent that was born abroad (Keij 2000).

Self-perceived general health was measured with a four-point ordinal scale and dichotomized into good health versus less than good health (Ware et al. 1996). Information was also collected on time to pregnancy (in months), parity, weeks of gestation, birth weight (grams), folic acid supplements, contraceptive pill use before the last pregnancy, and whether the pregnancy was induced by assisted reproduction technologies (ART). Infants were defined as small for gestational age (SGA) when their birth weight was more than two standard deviations below the reference value for their gestational age (Usher and McLean 1969). Preterm delivery was defined as a birth before 35 weeks of gestation (10th percentile).

We ascertained dietary patterns during the first 6 months of pregnancy. One general question distinguished vegetarian diets and diets rich in vegetables, fruits, meat, or fish. A phytoestrogen-specific food questionnaire was developed to differentiate categories of exposure based on a semiquantitative estimation of the intake of food products containing isoflavonoids and lignans, which are considered the most important naturally occurring phytoestrogens. The questionaire was developed for this study by TNO Food and Nutrition Research (Zeist, the Netherlands; Brants 1999). For the questionnaire, food products were selected that may contribute to isoflavonoid or lignan intake based on previous research (Brants 1999). For soy consumption, all known soy products were selected except soy oil and soy sauce, because they contain little or no biologically active isoflavonoids. Lignan-containing products were selected for their contribution to the total lignan intake, which was estimated on the basis of their lignan contents (according to food-constituent tables) and the use of the product in the general population (including nonusers) or in the group of users (Brants 1999). We also considered the feeding patterns in Surinam, Turkish, or Moroccan culture (the main groups of immigrants in Rotterdam). We quantified the average daily intake of phytoestrogens (based on consumption per week) by multiplying frequency of use by portion size by the concentration of phytoestrogens according to food-constituent tables (Brants 1999). The intakes per product were added up to the total intake of lignans and isoflavonoids to allow differentiation of subjects with high, intermediate, and low intake, based on tertiles.

We derived occupational exposure from generic questions on paid employment (yes/no) and jobs held in the year before delivery. The focus was on chemicals that may have endocrine activity (Van Tongeren et al. 2002) or that have previously been described as male reproductive toxicants (Tielemans et al. 1999a). For the few parents with multiple jobs, the job with most working hours was selected at the time of the first trimester (for mothers) or around fertilization (for fathers). Parents without a job were considered as having no occupational exposures. Additional questions were asked about job title, type of business, name of employer, and activities in the job. A checklist was used for self-reported exposure (yes/no) to ionizing radiation, physical exposures, and classes of chemical substances that have been linked to human reproductive impairment, such as solvents, pesticides, and heavy metals (Tielemans et al. 1999a). Subjects were classified as being exposed to solvents when reporting contact in their job to industrial cleaning products (degreasers), paints, printing inks, glues, or industrial cleaning products (Tielemans et al. 1999b).

We also assessed occupational exposure by applying a job-exposure matrix (JEM) for potential EDs (Van Tongeren et al. 2002). The JEM was based on the judgment of occupational hygienists who estimated for particular jobs the exposure to seven categories of potential EDs (e.g., pesticides and polychlorinated organic compounds) (Van Tongeren et al. 2002). A person in a particular job was assigned “probable exposure = yes” if the experts judged that it was probable that a reasonable proportion of workers had some exposure. An overall classification of “probable exposure to potential EDs = yes” was given to a job if at least one of the seven exposure categories was scored as “yes.”

### Statistics.

The agreement between self-reported exposure and exposure classification derived from the JEM was determined by the weighted Cohen’s κ. A κvalue < 0.4 was considered poor agreement, 0.4–0.6 moderate agreement, and > 0.6 good agreement (Landis and Koch 1977).

We computed frequency counts, crude odds ratios (ORs), and 95% confidence intervals (95% CIs) for all potential risk factors. Continuous risk factors were categorized into three or four categories for ease of interpretation. Trends were assessed by a chi-square test for trends in 2 × 3 or 2 × 4 tables. Logistic regression analysis with stepwise forward selection on univariate risk factors was used to arrive at a multivariable model for either outcome, with a significance level of 0.05 for retained variables. In addition, exposure variables of interest were also included in a multivariable model when this factor was statistically significantly associated with either cryptorchidism or hypospadias in the univariate analysis and the factor caused a change by ≥ 15% in the coefficient of other risk factors in the model. Interactions of all variables were also tested for significance. The 95% CIs around the ORs were derived from the individual Wald’s statistics, except for variables with cell frequencies of five or fewer, in which case likelihood-based confidence intervals are given. Because information on fathers was not collected on all children, we performed separate analyses for those with mother information and those with mother and father information. Regression analyses were performed using PROC LOGISTIC in SAS (version 8.2; SAS Institute, Cary, NC, USA).

## Results

The general characteristics of the study population are shown in Tables 1 and 2. Table 1 presents the risk factors for cryptorchidism and hypospadias related to the mother and pregnancy. Significant risk factors were related to intrauterine growth (low birth weight and SGA for hypospadias, preterm delivery for cryptorchidism). Mothers with better general health, higher education, and larger height showed less risk of having offspring with either abnormality. These individual characteristics were strongly interrelated. Boys born from mothers of Turkish origin had increased risks for cryptorchidism and hypospadias. Compared with a Dutch origin, a Turkish origin was strongly associated with suboptimal maternal health, a lower education level, and lower maternal height. Dietary phytoestrogens and maternal occupational exposure to potential EDs did not significantly alter the risk of either abnormality.

Table 2 presents paternal risk factors. Paternal age, education, and country of origin were associated with cryptorchidism and hypospadias. Smoking among fathers was associated with hypospadias (OR = 3.4). ORs for cryptorchidism in offspring were elevated for self-reported solvent exposure (OR = 2.0) and pesticide exposure according to the JEM (OR = 4.5). Self-reported exposure to pesticides also gave an increased risk (OR = 2.8) of borderline significance (*p* = 0.08). Paternal self-reported solvent exposure (OR = 2.4) was also associated with hypospadias. Self-reported exposure to heavy metals, anesthetics, and other JEM categories was not significantly associated with the outcomes.

The exposure prevalence in men was significantly higher than in women. Among men, the prevalence of self-reported exposure was 23.0% (*n* = 75) for solvents, 10.2% (*n* = 33) for heavy metals, 4.6% (*n* = 15) for pesticides, and 1.9% (*n* = 6) for anesthetics, and 31% were exposed to at least one of these categories. The single largest group reporting pesticide exposure were workers in greenhouses involved in cultivation of vegetables (*n* = 3) or flowers (*n* = 3). The JEM identified paternal ED exposure in 12.0% of the fathers. In the JEM, pesticide exposure (*n* = 14) was assigned primarily to greenhouse workers in flowers (*n* = 7) or vegetables (*n* = 6). Among couples, maternal and paternal exposures to pesticides were associated for self-reports and the JEM (Spearman rank correlation, 0.18 and 0.21, respectively). The agreement between pesticide exposures based on self-reports and the JEM was moderate (κ= 0.54; 95% CI, 0.36–0.71). Age, education level, smoking, and country of origin within couples were strongly correlated (Spearman correlation coefficients > 0.50).

Tables 3 and 4 present the multivariate models with maternal and paternal risk factors for cryptorchidism and hypospadias, respectively. The final models on maternal risk factors (Tables 3 and 4) provide no evidence for an association between maternal dietary and environmental exposure and the occurrence of both outcomes while adjusting for other risk factors. When manually added to the final multivariate models, the risk estimates for occupational exposures and dietary phytoestrogens were very similar to their effects in the univariate analyses in Table 1 (< 15% change in coefficient), although the confidence intervals were somewhat larger.

A preterm delivery and a low education level were the strongest risk factors for cryptorchidism in the maternal multivariate model, together with an interaction between country of origin and mother’s age at delivery. Among Turkish mothers ≥ 30 years of age, an increased risk of cryptorchidism in newborns was observed compared with younger Turkish and with Dutch mothers. When taking into account also the characteristics of the father (Table 3), the only paternal risk factor associated with cryptorchidism was probable occupational exposure to pesticides (OR = 3.8). Although not selected by the stepwise forward selection, manual addition of self-reported exposure to solvents produced a similar effect as in the univariate analysis (OR = 1.9; 95% CI, 0.9–3.9), but the influence of probable exposure to EDs was substantially smaller (OR = 1.3; 95% CI, 0.5–3.3) than when analyzed univariately.

The important maternal risk factors for hypospadias were SGA birth and health status of the mother (Table 4). Again, Turkish origin was associated with an increased risk for hypospadias (OR = 3.0), but no interaction with age was identified. When also taking into account the characteristics of the father, current smoking of the father was a strong risk factor (OR = 3.8). The risk for self-reported exposure to solvents among fathers was elevated (OR = 2.0) but of borderline significance (*p* = 0.09). This risk factor was included because it influenced the risk estimates of time to pregnancy, because of the moderate association between time to pregnancy and solvent exposure. When manually entered into the multivariate model, the risk estimates for maternal and paternal occupational exposures and dietary phytoestrogens (that were not selected by the stepwise procedure) were very similar to their univariate effects, except for a reduced risk associated with self-reported maternal exposure to pesticides (OR = 1.1; 95% CI, 0.2–6.2) and an increased risk associated with lignan intake of 4–6 g/day (OR = 1.5; 95% CI, 0.6–3.5) and < 4 g/day (OR = 1.7; 95% CI, 0.7–3.9).

## Discussion

This study reports the novel findings that paternal pesticide exposure is a risk factor for cryptorchidism and that paternal smoking is associated with hypospadias in the offspring. A strength of the study is that the results are based on a case–control study nested within a large birth cohort in the general population of Rotterdam. Because 95% of all consecutive newborn boys in Rotterdam were prospectively subjected to a standardized examination of the external genitalia, bias in case identification by exposure is unlikely. The prevalence of 1.1% cryptorchidism and 0.8% hypospadias in our population has been described elsewhere and is within the range reported by comparable studies (Pierik FH et al., unpublished data; Pierik et al. 2002). A good accuracy of the diagnosis of both abnormalities by CHC physicians is expected because of the standardized and systematic examination of a birth cohort. A high accuracy (88% verification) of the hypospadias diagnosis by CHC physicians has been demonstrated previously (Pierik et al. 2002), whereas the accuracy of cryptorchidism diagnosis was not assessed. Because the case status was assessed prospectively before data on determinants were collected, the misclassification by CHC physicians is probably nondifferential, which would bias the results toward unity in our analyses. Resources were insufficient to have CHC physicians report the exact location of the urethral opening and the left and right testis for the nearly 9,000 subjects. Another strength of the present study is that both maternal and paternal determinants were included. A weakness of the study is that the paternal determinants were missing for 26% (*n* = 116) of the subjects, and in the subjects with paternal information, the paternal determinants were presented by the fathers themselves in only 28% (*n* = 91). Differential misclassification between mothers and fathers on self-reported paternal exposure to solvents cannot be ruled out because fathers and mothers reported a paternal exposure prevalence of 31 and 20%, respectively. However, the hypospadias risk for paternal solvent exposure reported by the father (OR = 1.9; 95% CI, 0.6–6.2) or mother (OR = 2.5; 95% CI, 1.0–6.2) was comparable in size, although the 95% CI was wider in these smaller subsets. For other paternal occupational exposures and lifestyle factors, such as smoking and alcohol use, no differences were observed between reporting mothers and fathers.

The multivariate analyses suggest an important role of paternal smoking and occupational exposures. Paternal smoking was significantly associated with hypospadias (OR = 3.8; Table 4). Paternal smoking has previously been associated with the occurrence of single and multiple birth defects (Zhang et al. 1992), but not specifically with hypospadias. Paternal smoking could have an effect through passive exposure of the mother, but this is unlikely because active smoking by the mother was not a risk factor. We cannot exclude that mothers have underreported their smoking. When mothers of cases under-report their own smoking more than that of their partner, paternal smoking may partly be a spurious risk factor.

After correction for other significant risk factors, paternal pesticide exposure based on the JEM was significantly associated with cryptorchidism (OR = 3.8; Table 3), and self-reported paternal solvent exposure was borderline associated with hypospadias (OR = 2.0; Table 4). The exposure classifications of solvents and pesticides were too broad to allow identification of specific (groups of) chemical agents to be held responsible for the increased risks of either anomaly. Because parents of cryptorchidism and hypospadias cases may have been more concerned with and knowledgeable about environmental risk factors than were parents of controls, differential reporting between cases and controls may have occurred. However, several reasons argue against information bias explaining the observed associations. First, the increased cryptorchidism risk for self-reported pesticide exposure (OR = 2.8) was confirmed by the independent JEM-based pesticide exposure (OR = 4.5). Unfortunately, no JEM judgment was available to validate self-reported solvent exposure. Second, parents were not informed about potential risk factors or the JEM classification. Third, the agreement between self-reported and JEM exposures was not different between cases and controls.

The JEM was developed for a study on occupational risk factors for hypospadias, with a focus on EDs (Van Tongeren et al. 2002; Vrijheid et al. 2003). The interexpert agreement among the industrial hygienists developing the JEM was good for pesticides (κ= 0.77) (Van Tongeren et al. 2002). Although the JEM may misclassify occupational exposures, nondifferential misclassification leads to attenuation of the ORs when both the outcome and the determinant are dichotomous variables (Chen 1989; Greenland 1980), and cannot explain the observed association between cryptorchidism and JEM-based pesticide exposure.

Some studies have reported on the association between occupational exposure and birth defects. Paternal solvent exposure has been associated with cleft palate, neural tube defects, and preterm birth (Kristensen et al. 1993; Olshan et al. 1991). A study among gardener and farmer families applying pesticides reported an increased risk of cryptorchidism and hypospadias in their offspring (Kristensen et al. 1997) but could not distinguish paternal from maternal exposure. Another study observed an increased risk of cryptorchidism in sons of female gardeners and farmers but not in sons of men working in farming or gardening (Weidner et al. 1998). Neither paternal nor maternal occupation was associated with hypospadias. Because exposure assessment was limited to job title, limited information was available on the role of specific occupational exposures, such as pesticide use (Weidner et al. 1998).

It remains to be established whether the associations between external agents and cryptorchidism and hypospadias are causal or based on confounding (e.g., by unknown but related occupational risk factors). Several plausible biologic mechanisms that could mediate the observed effects of paternal smoking and occupational exposure on the offspring have, however, been described. There is growing human evidence that paternal environmental factors around the time of fertilization play a role after fertilization. More than 100 chemicals, including pesticides and solvents, have been related to male-mediated adverse reproductive outcomes (Davis et al. 1992). Animal studies provide extensive evidence for male-mediated developmental effects (i.e., spontaneous abortions, growth retardation, malformations, and behavioral abnormalities) of environmental agents (Robaire and Hales 2003). Several modes of action of chemicals have been shown, the most likely being genetic (e.g., germline DNA modification) or epigenetic (e.g., DNA repair, chromatin structure, apoptosis) effects on germ cells, whereas exposure of the oocyte or embryo to contaminated seminal fluid could also play a role (Davis et al. 1992; Robaire and Hales 2003). A study in mice demonstrated that environmental pollution resulted in DNA mutations that were inherited by the offspring, primarily through the paternal germline (Somers et al. 2002).

On the basis of the xenoestrogen hypothesis (Sharpe 2003), we anticipated that maternal exposure to EDs during fetal life could be a causal pathway leading to cryptorchidism and hypospadias. As of yet, few human data are available to confirm or refute this hypothesis. We did not find an association between maternal occupational exposure and either abnormality, perhaps due to the small proportion of exposed mothers. A previous study reported a maternal vegetarian diet as a risk factor for hypospadias and suggested a higher phytoestrogen intake as explanation (North and Golding 2000). We specifically assessed dietary phytoestrogen intake, which was not a significant risk factor for hypospadias or cryptorchidism. However, the nutrition data may suffer from inaccuracies because nutrition was assessed only once, whereas considerable intraindividual variation has been described with food-frequency questionnaires (Goldbohm et al. 1995). The findings in our case–control study suggest an association between cryptorchidism and hypospadias and lower socioeconomic status, as reflected in low education level and suboptimal general health status of both parents. The effect of socioeconomic status may be confounded by selection bias, especially because of differential response between cases and controls. For the impact of education to be spurious, this would require approximately a 2-fold higher response among parents of cases than of controls in subjects with a low education.

A similar differential response bias may have contributed to the observed effect of Turkish origin on cryptorchidism and hypospadias. Based on the nationalities of all 8,695 examined boys, Moroccan, Turkish, and other minorities were underrepresented by about 40–50% among controls. To exclude confounding by country of origin, we repeated the regression analysis in Dutch subjects only, which did not yield significantly different results, although standard errors increased because of a smaller sample. Among Dutch subjects paternal exposure to pesticides has a similar effect (OR = 3.4; 95% CI, 0.3–43.0) on cryptorchidism but failed to reach the level of conventional significance. Paternal smoking (OR = 6.5; 95% CI, 2.0–21.7) and self-reported paternal exposure to solvents (OR = 3.3; 95% CI, 1.2–9.5) remained significant risk factors for hypospadias among Dutch subjects.

Previous studies have reported ethnic variations in the occurrence of cryptorchidism and hypospadias (Chia et al. 2003; Fredell et al. 2002). Familial aggregation has been described for both abnormalities, supporting the importance of genetic factors (Fredell et al. 2002; Weidner et al. 1999). The association between Turkish origin and cryptorchidism and hypospadias may be the result of a genetic or environmental factor among Turkish people that predisposes toward these abnormalities. A higher maternal age was a significant risk factor within the Turkish minority, but not in the overall group of non-Turkish origin. We cannot exclude the possibility that the response may have been different with age among Turks.

In the multifactorial models without adding paternal risk factors, preterm delivery was associated with cryptorchidism (OR = 3.1; Table 3), and being SGA was associated with hypospadias (OR = 7.3; Table 4). These associations are well known from previous studies (Weidner et al. 1999). Some authors point to reduced placental function as underlying etiology for low birth weight, cryptorchidism, and hypospadias (Fredell et al. 1998).

Some earlier studies looking at large groups of cases have reported ORs ranging from 1.1 to 1.9 for low birth order and a higher maternal age as risk factors for cryptorchidism or hypospadias cases (Akre et al. 1999; Biggs et al. 2002; Kallen 2002; Møller and Skakkebaek 1997), although others did not observe these excess risks (Berkowitz et al. 1995; Jones et al. 1998). Birth order and parental age were not significantly related to cryptorchidism or hypospadias in our study, which may be because of the relatively small effect and limited population size.

Our observation that a longer time to pregnancy was associated with hypospadias (Table 4) may be explained by familial aggregation of hypospadias (Fredell et al. 2002) and its association with subfertility (Skakkebaek et al. 2001). Previous studies have reported a higher incidence of hypospadias in boys born after intracytoplasmic sperm injection (Ericson and Kallen 2001; Wennerholm et al. 2000), which may be explained by a lower birth weight that occurs more frequently after ART. In our study, the frequency of ART was too low to evaluate its association with hypospadias or cryptorchidism.

This study suggests that paternal environmental exposures may increase the risk of cryptorchidism and hypospadias in newborn boys, which may indicate an effect on the paternal germline. Cryptorchidism was associated with paternal exposure to pesticides, and hypospadias was more frequent in fathers that were active smokers. The pregnancy-related risk factors of low birth weight and SGA birth for hypospadias and preterm delivery for cryptorchidism have consistently been found in previous studies (Weidner et al. 1999). Future studies on environmental risk factors for cryptorchidism and hypospadias should not only focus on maternal exposure during fetal life but also include the paternal pathway to substantiate whether the observed associations are causal.

## Figures and Tables

**Table 1:** Univariate analysis of the association between maternal risk factors and the occurrence of cryptorchidism and hypospadias in a case–control study among 443 mother–child pairs.

		Cryptorchidism (*n* = 78)		Hypospadias (*n* = 56)	
Variable	Controls	Cases	OR (95% CI)	Cases	OR (95% CI)
Age at delivery (years)					
< 25	48	14	1.0	9	1.0
25–30	80	20	0.9 (0.4–1.9)	17	1.1 (0.5–2.7)
30–35	111	29	0.9 (0.4–1.8)	19	0.9 (0.4 –2.2)
≥35	70	15	0.7 (0.3–1.7)	11	0.8 (0.3–2.2)
Height (cm)					
< 160	41	16	1.0**	16	1.0**
160–165	65	25	1.0 (0.5–2.1)	10	0.4* (0.2–1.0)
165–170	95	14	0.4* (0.2–0.9)	12	0.3* (0.1–0.8)
≥170	111	23	0.5 (0.3–1.1)	18	0.4* (0.2–0.9)
Education level					
Low	65	27	1.0	21	1.0
Intermediate	154	37	0.6 (0.3–1.0)	23	0.5* (0.2–0.9)
High	94	14	0.4* (0.2–0.7)	12	0.4* (0.2–0.9)
Country of origin					
Netherlands	170	34	1.0	28	1.0
Morocco	21	8	1.9 (0.8–4.7)	3	0.9 (0.2–2.7)
Turkey	18	15	4.2* (1.9–9.1)	8	2.7* (1.0–6.6)
Surinam	35	8	1.1 (0.5–2.7)	5	0.9 (0.3–2.2)
Other	69	13	0.9 (0.5–1.9)	12	1.1 (0.5–2.2)
Good general health					
Yes	291	66	1.0	43	1.0
No	22	12	2.4* (1.1–5.1)	13	4.0* (1.9–8.5)
Current smoker					
Yes	71	22	1.3 (0.8–2.3)	18	1.6 (0.9–3.0)
No	242	56	1.0	38	1.0
ART					
Yes	14	4	1.2 (0.3–3.3)	3	1.2 (0.3–3.9)
No	299	74	1.0	53	1.0
Time to pregnancy					
0 months	96	26	1.0	13	1.0
1–3 months	113	21	0.7 (0.4–1.3)	24	1.6 (0.8–3.3)
≥4 months	91	26	1.1 (0.6–2.0)	15	1.2 (0.6–2.7)
Birth weight (g)					
< 3,000	57	19	1.5 (0.7–3.0)	21	4.1* (1.7–9.8)
3,000–3,500	106	26	1.1 (0.6–2.1)	15	1.6 (0.6–3.8)
3,500–3,750	58	11	0.8 (0.4–1.9)	9	1.7 (0.6–4.7)
≥3,750	88	20	1.0	8	1.0**
SGA					
Yes	7	2	1.2 (0.2–4.9)	6	5.5* (1.8–17.1)
No	302	74	1.0	47	1.0
Premature birth					
Yes	25	14	2.5* (1.2–5.1)	8	1.9 (0.8–4.5)
No	288	64	1.0	48	1.0
Primiparous					
Yes	162	44	1.2 (0.7–2.0)	25	0.8 (0.4–1.3)
No	151	34	1.0	31	1.0
Folic acid supplements in pregnancy					
Yes	179	35	0.6 (0.4–1.0)	32	1.0 (0.6–1.8)
No	134	43	1.0	24	1.0
Vegetable-rich diet					
Yes	125	24	0.7 (0.4–1.1)	17	0.7 (0.4–1.2)
No	186	54	1.0	39	1.0
Soy protein intake					
≥20 g/day	51	8	0.6 (0.3–1.3)	9	1.0 (0.5–2.2)
> 0–20 g/day	41	12	1.1 (0.6–2.3)	8	1.1 (0.5–2.5)
0 g/day	221	58	1.0	39	1.0
Lignan intake					
≥6 g/day	115	23	0.7 (0.4–1.3)	22	1.0 (0.5–2.1)
4–6 g/day	119	31	0.9 (0.5–1.6)	19	0.8 (0.4–1.8)
< 4 g/day	79	24	1.0	15	1.0
Paid employment					
Yes	213	46	0.7 (0.4–1.1)	31	0.6 (0.3–1.0)
No	100	32	1.0	25	1.0
Probable exposure to EDs (JEM)					
Yes	24	6	1.0 (0.4–2.6)	3	0.7 (0.2–2.0)
No	289	72	1.0	53	1.0
Probable exposure to pesticides (JEM)					
Yes	7	2	1.2 (0.2–4.9)	2	1.6 (0.2–6.9)
No	306	76	1.0	54	1.0
Self-reported exposure to pesticides					
Yes	4	2	2.0 (0.3–10.6)	1	1.4 (0.1–9.7)
No	309	76	1.0	55	1.0
Self reported exposure to solvents					
Yes	32	6	0.7 (0.3–1.8)	9	1.7 (0.8–3.8)
No	281	72	1.0	47	1.0

* *p* < 0.05.

** **Significant trends were observed for maternal height with cryptorchidism and hypospadias (OR = 0.67 and 0.52 per 10 cm height increase, respectively) and birth weight and hypospadias (OR = 0.91 per 100 g of body weight increase).

**Table 2:** Univariate analysis of the association between paternal risk factors and the occurrence of cryptorchidism and hypospadias in a case–control study among 326 father–child pairs.

		Cryptorchidism (*n* = 50)		Hypospadias (*n* = 41)	
Variable	Controls	Cases	OR (95% CI)	Cases	OR (95% CI)
Age (years)					
< 25	19	10	1.0	5	1.0
25–30	43	6	0.3* (0.1–0.8)	12	1.1 (0.3–3.4)
30–35	64	19	0.6 (0.2–1.4)	8	0.5 (0.1–1.6)
> 35	109	15	0.3* (0.1–0.7)	16	0.6 (0.2–1.7)
Height (cm)					
< 175	59	14	1.0	12	1.0
175–180	42	9	0.9 (0.4–2.3)	5	0.6 (0.1–1.8)
180–185	48	12	1.1 (0.5–2.5)	11	1.1 (0.5–2.8)
> 185	82	15	0.8 (0.4–1.7)	14	0.8 (0.4–2.0)
Educational level					
Low	59	19	1.0	12	1.0
Intermediate	89	13	0.5* (0.2–1.0)	25	1.4 (0.6–4.0)
High	85	18	0.7 (0.3–1.4)	5	0.3* (0.1–0.9)
Country of origin					
Netherlands	127	26	1.0	25	1.0
Morocco	16	6	1.8 (0.6–4.9)	2	0.6 (0.1–2.4)
Turkey	16	11	3.4* (1.4–8.1)	7	2.2 (0.8–5.8)
Surinam	31	2	0.3 (0.1–1.1)	3	0.5 (0.1–1.5)
Other	46	5	0.5 (0.2–1.4)	5	0.6 (0.2–1.4)
Good general health					
Yes	205	39	1.0	34	1.0
No	30	10	1.8 (0.8–3.9)	7	1.4 (0.6–3.5)
Current smoker					
Yes	98	22	1.2 (0.6–2.1)	29	3.4* (1.7–7.0)
No	138	27	1.0	12	1.0
Paid employment					
Yes	209	41	0.7 (0.3–1.6)	37	1.0 (0.3–3.6)
No	27	8	1.0	5	1.0
Probable exposure to potential EDs (JEM)					
Yes	38	13	1.8 (0.9–3.8)	10	1.6 (0.7–3.6)
No	198	37	1.0	32	1.0
Probable exposure to pesticides (JEM)					
Yes	7	6	4.5* (1.4–13.9)	1	0.8 (0.3–3.6)
No	229	44	1.0	41	1.0
Self-reported exposure to pesticides					
Yes	9	5	2.8 (0.8–8.5)	1	0.6 (0.0–3.4)
No	227	45	1.0	41	1.0
Self-reported exposure to solvents					
Yes	45	16	2.0* (1.0–3.9)	15	2.4* (1.2–4.8)
No	191	34	1.0	27	1.0

* *p* < 0.05.

**Table 3:** Multivariate models of the association between maternal and paternal risk factors and the occurrence of cryptorchidism in a case–control study.

Risk factors	OR (95% CI)
Maternal risk factors (*n* = 443)	
Education level (low vs. intermediate/high)	1.9* (1.0–3.4)
Premature birth ( > 2 weeks)	3.1* (1.5–6.6)
Interaction age at delivery and country of origin:	
Non-Turkish mothers < 30 years of age	1.0
Turkish mothers < 30 years of age	2.0 (0.7–5.6)
Non-Turkish mothers ≥ 30 years of age	0.8 (0.5–1.5)
Turkish mothers ≥ 30 years of age	16.3* (3.3–81.2)
Maternal and paternal risk factors (*n* = 326)	
Good general health of mother (no vs. yes*^a^*)	3.8* (1.5–9.8)
Vegetable-rich diet of mother (yes vs. no*^a^*)	0.4* (0.2–0.9)
Probable exposure to pesticides of father (JEM)	3.8* (1.1–13.4)
Interaction age at delivery and country of origin:	
Non-Turkish mothers < 30 years of age	1.0
Turkish mothers < 30 years of age	1.6 (0.5–5.6)
Non-Turkish mothers ≥ 30 years of age	1.0 (0.5–2.0)
Turkish mothers ≥ 30 years of age	8.8* (1.2–63.2)

a Reference.

* *p* < 0.05.

**Table 4:** Multivariate models of the association between maternal and paternal risk factors and the occurrence of hypospadias in a case–control study.

Risk factors	OR (95% CI)
Maternal risk factors (*n* = 443)	
Education level (low vs. intermediate/high)	2.0* (1.1–3.9)
SGA (yes vs. no)	4.2* (1.2–14.7)
Turkish origin of mother (vs. non-Turkish)	3.0* (1.2–7.7)
Good general health (no vs. yes*^a^*)	3.6* (1.6–8.1)
Maternal and paternal risk factors (*n* = 326)	
SGA (yes vs. no*^a^*)	7.3* (1.7–31.4)
Current smoker, father (yes vs. no*^a^*)	3.8* (1.8–8.2)
Self-reported exposure to solvents of father	2.0 (0.9–4.6)
Time to pregnancy	
0 months	1.0
1–3 months	3.9* (1.3–11.5)
≥4 months	3.4* (1.1–10.3)

a Reference.

* *p* < 0.05.
